# Effects of molecular weight on intestinal anti-inflammatory activities of β-D-glucan from *Ganoderma lucidum*

**DOI:** 10.3389/fnut.2022.1028727

**Published:** 2022-09-29

**Authors:** Yanfang Liu, Qingjiu Tang, Jie Feng, Jing Liu, Chuanhong Tang, Mengqiu Yan, Shuai Zhou, Liping Liu, Jing Zhou, Jingsong Zhang

**Affiliations:** ^1^Institute of Edible Fungi, Shanghai Academy of Agricultural Sciences, Ministry of Agriculture, Shanghai, China; ^2^Key Laboratory of Edible Fungi Resources and Utilization (South), Ministry of Agriculture, Shanghai, China; ^3^National Engineering Research Center of Edible Fungi, Shanghai, China; ^4^Shanghai Baixin Bio-Tech Co., Ltd., Shanghai, China

**Keywords:** *Ganoderma lucidum*, β-(1 → 3, 1 → 6)-D-glucan, molecular weight, inflamed Caco-2 cells, DSS-induced colitis, inflammatory cytokines

## Abstract

To investigate the influence of molecular weight (*M*_w_) on the anti-inflammatory activity of β-D-glucan from *Ganoderma lucidum*, ultrasonic irradiation was applied to treat the β-D-glucan (GLP, 2.42 × 10^6^ g/mol) solution to obtain two degraded fractions with molecular weight of 6.53 × 10^5^ g/mol (GLPC) and 3.49 × 10^4^ g/mol (GLPN). Structural analysis proved that the degraded fractions possessed similar repeated units with the original β-D-glucan. The *in vitro* anti-inflammatory activity studies showed that all fractions could significantly inhibit LPS-induced expression of cytokines including TNF-α, IL-8, MIF and MCP-1 in Caco-2 cells at certain concentrations. Moreover, GLPC and GLPN exhibited better anti-inflammatory activity than GLPC. The intestinal anti-inflammatory activity evaluated by dextran sulfate sodium (DSS)—induced colitis mice model showed that intragastric administration of GLPN (lower *M*_*w*_ fraction) could significantly recover inflamed tissues of mice. Compared with GLP and GLPC, GLPN exhibited stronger ability to inhibit the secretion of pro-inflammatory cytokines (TNF-α, IL-1β, and IL-6). The results revealed that *M*_*w*_ of β-D-glucan influenced its anti-inflammatory activity and decreasing of *M*_*w*_ would improve the activity, which provided evidence for the potential use of β-D-glucan from *G. lucidum* as anti-colitis ingredients.

## Introduction

In recent years, inflammatory bowel disease (IBD) has gradually become a common disease in the world with the increase in the consumption of high-fat and high-sugar diets (1). IBD, including Crohn's disease (CD) and ulcerative colitis (UC), is characterized by chronic inflammatory disorders of the gastrointestinal tract without clearly defined causes (2). IBD disease had a significant impact on quality of life due to some ongoing symptoms including reduced ability to work, social stigma, management of toilet access issues and difficulty with physical intimacy. Although the exact pathogenesis of IBD is not clear, it is generally accepted that the uncontrolled immune responses lead to intestinal inflammation, which is associated with the increase of pro-inflammatory cytokines including tumor necrosis factor-α (TNF-α) and interleukins such as Interleukin-1β (IL-1β), Interleukin-6 (IL-6), and Interleukin-8 (IL-8) in the intestine (3, 4). Hence, the priority for the therapy is to regulate the immune disorders by suppressing the levels of inflammatory mediators to control the progression of IBD. In recent years, interest on the development of efficient new drugs or supplements derived from natural sources for the treatment of IBD is growing due to their efficacy and safety (5).

The polysaccharides obtained from natural resources including plants, animals and microorganisms have been found to exhibit the advantages of safety and high therapeutic efficacy, especially for regulating the immune system (6). Some researchers have discovered that several kinds of polysaccharides, such as pectin, chitosan and polysaccharides from microorganisms, could effectively cure UC inflammatory diseases (7–9). These polysaccharides showed effective influence on the treatment of UC *via* the regulation of inflammatory cytokines, intestinal flora and immune system (6), which provided some alternatives for the treatment of inflammatory bowel disease.

β-glucans, the main active polysaccharides from natural resources, especially those from fungi, have exhibited several bioactivities including anti-cancer, immune-modulating and anti-inflammatory properties (1, 10). These β-glucans normally had β-(1 → 3)-linked D-glucose as the backbone with various β-(1 → 6)-D-glucopyranosyl branching units. The difference in sources and preparation methods can affect the physicochemical features of polysaccharides, such as the solubility, degree of branching, molecular weights and conformational structures, which might significantly influence their activities (11–14). Recently, several studies demonstrated that oral administration of β-glucans from yeast or mushrooms exhibited anti-inflammatory effects on dextran sulfate sodium (DSS)-induced colitis in mice (7, 9, 15), which indicated that β-glucans from yeast or mushrooms might be effective drugs or healthcare products to prevent and treat UC in clinical application. Previous studies revealed that the molecular weight (*M*_*w*_) of polysaccharides was associated with their physiological characteristics and biological activities (16, 17). It was reported that molecular weight showed obvious influence on the antitumor and immunological activities of polysaccharides (18–20).

*Ganoderma lucidum* (*G. lucidum*), a famous medicinal mushroom used as traditional Chinese medicine for centuries in China, has been reported to contain many kinds of bioactive compounds which could stimulate the immune system and promote health and longevity. As one of the main polysaccharides, β-D-glucan has been extracted and purified from *G. lucidum* and exhibited many pharmacological activities, especially for immunoregulation (21–23). Our previous research also indicated that the molecular weight of β-(1 → 3)-D-glucan with β-(1 → 6) branches from *G. lucidum* had impacts on the immune-enhancing activity, and the fractions with high *M*_*w*_ (>1.8 × 10^6^ g/mol) exhibited better activity (21). However, the influence of *M*_*w*_ on anti-inflammatory activity of β-D-glucan was not investigated well. Moreover, the comprehension of the relationship between *M*_*w*_ and anti-inflammatory activities of β-D-glucan from *G. lucidum* is necessary for their further application. It has been reported that ultrasonic degradation was a useful physical method for producing polymers with lower *M*_*w*_, and the chemical structure of the polymer could be maintained during the degradation process (24, 25). In order to investigate the association of *M*_*w*_ and anti-inflammatory activities of β-D-glucan from *G. lucidum*, ultrasonic treatment was performed to obtain two β-D-glucan fractions with lower *M*_*w*_, and the anti-inflammatory effects of β-D-glucans with different *M*_*w*_ were compared. Furthermore, the anti-inflammatory activities of β-D-glucans were evaluated through a DSS-induced colitis mice model, which could be useful for further study and application of β-D-glucan from *G. lucidum*.

## Materials and methods

### Materials

*Ganoderma lucidum* fruit bodies (cultivar longzhi No. 2) were cultivated and collected from Zhejiang province in China. Human epithelial colorectal adenocarcinoma (Caco-2) cells line was purchased from the cell bank at the Chinese Academy of Sciences. Minimum essential medium (MEM) and fetal bovine serum (FBS) were from Invitrogen-Gibco (New York, USA). Human cytokine ELISA kits including TNF-α, IL-8, MIF, and MCP-1were from Beijing 4A Biotech Co., Ltd (Beijing, China). Mouse cytokine ELISA kits including TNF-α, IL-1β, and IL-6 were purchased from Shanghai Jiake Bio. Tech. Co., Ltd (Shanghai, China). Dextran sulfate sodium (DSS, *M*_*w*_: 36,000–50,000 g/mol) was purchased from MP Biomedicals LLC (California, USA). Dextran (*M*_*w*_, 80,000 g/mol) was from Sigma-Aldrich (Missouri, USA). All other reagents except otherwise specified were analytical grade and from Chinese sources.

### Animals

C57BL/6 mice (4 weeks of age, male) were from Shanghai SLAC Laboratory Animal Co., Ltd. Animal quality certificate number: SCXK (Hu) 2017-0005. The animals were housed in plastic cages under normal feeding conditions. Every experimental protocol involving the animals was performed according to the line of legislation and ethical guidelines of the People's Republic of China, and was approved by the Ethics Committee of Experimental Animal Care at Shanghai University of Traditional Chinese Medicine (Permit No. PZSHUTCM210926015).

### Preparation of β-D-glucan with different molecular weights

β-D-glucan (GLP) was isolated and purified from hot water extracts of *Ganoderma lucidum* fruit bodies by 20% (v/v) ethanol precipitation according to the previous article (21). The total sugar content was determined to be 96.1% based on the phenol-sulfuric acid method. GLP was suspended in distilled water and stirred under 80°C for several hours to obtain the solution with a concentration of 2 mg/mL. Then GLP solution was treated with 20 kHz ultrasonic irradiation at 900 W using an ultrasonic reactor (JY-99 II, Ningbo Xin Zhi Biotechnology Co., Ltd., China) for 2 h under a controlled temperature lower than 50°C. After being centrifuged at 8,000 × *g* for 15 min, the supernatant was collected and freeze-dried to obtain the degraded fraction GLPC. GLPC solution (2 mg/mL) was continually treated with ultrasonic irradiation at 1,200 W for 2 h under a controlled temperature lower than 50°C. After being centrifuged at 8,000 × *g* for 15 min, the supernatant was collected and freeze-dried to obtain the degraded fraction GLPN. The *M*_*w*_ and characteristics of three fractions were analyzed below.

### Molecular weight distribution and characteristics analysis

2–5 mg samples (GLP, GLPC, and GLPN) were weighed in tube and dissolved in 1 mL mobile solution containing 0.15 mol/L NaNO_3_, 0.05 mol/L NaH_2_PO_4_, and 0.02% NaN_3_ (pH 7.0), and the supernatant was collected for analysis after centrifugation. High-performance size exclusion chromatography (HPSEC) was applied to perform the molecular weight determination and conformational character evaluation. Among all the detectors, the eight-angle laser light scattering detector (MALLS, Wyatt Technology Corp, USA) was used for determining the absolute molar mass and size of polymers. The refractive index detector (RI, Waters Corporation, USA) was to assess the concentration of the solution. The online viscosity detector (VS, Wyatt Technology Corp, USA) was used for viscosity determination of the solution. Several SEC columns from TSK gel series (Japan) including a guard column, G6000 PW_XL_, G4000 PW_XL_, and G2500 PW_XL_ with different separation ranges of molecular weight distribution were connected to analyze the samples. The *dn/dc* value was set to 0.151 mL/g for polysaccharide polymers (21).

### Methylation and NMR analysis

In order to investigate the structure character and confirm that the original β-glucan and the degraded fractions possessed similar repeat units, the methylation analysis and 1D NMR spectra were performed in the study. Methylation analysis of three samples was performed based on the previous method (11), and the partially methylated alditol acetates (PMAA) were analyzed by a GC-MS system according to the reported procedures (12). GLP, GLPC and GLPN were dissolved in the mixture of Me_2_SO-*d6* and D_2_O (6:1, v/v) to a concentration of 30 mg/mL, respectively. 1D ^1^H and ^13^C NMR spectra were recorded at 70°C by using a NMR spectrometer (Bruker VNMRS 600, Germany).

### Inflamed Caco-2 cells model induced by LPS

Caco-2 cells were cultured in MEM containing 20% fetal bovine serum (FBS), penicillin (100 IU/mL) and streptomycin (100 μg/mL) in 5% CO_2_ at 37°C. The medium was renewed twice a week. The effect of samples on the viability of Caco-2 cells was evaluated by alamar blue assay. Briefly, cells were seeded at 1 × 10^5^ cells/well in a 96-well plate. After incubation for 24 h, the cells were treated with different concentrations of samples for 24 h. Then, 30 μL 0.01% alamar blue solutions were added and the cells were cultured for an additional 4–6 h. The cell viability was determined according to the instruction of the alamar blue assay.

To investigate the inhibition effect of samples on the pro-inflammatory cytokine expression levels in inflamed Caco-2 cells induced by LPS, 1 mL cells suspension (5 × 10^5^ cells/mL) was seeded in each well of 24-well plate, and 50 μL sample solutions (final concentrations of 10, 50, and 200 μg/mL) were added into each well. After being cultured for 2 h, the inflammation was induced by addition of 50 μL LPS (final concentration of 2 μg/mL). After being co-cultured for 24 h, the cells in each well were collected and homogenized in RIPA lysis buffer with protease inhibitor cocktail after 24 h (Thermo, USA). Then the homogenate was centrifuged at 3,000 × *g* for 10 min at 4°C, and the supernatant was diluted to the same final concentrations of protein based on the BCA assay (Thermo, USA) analysis. The pro-inflammatory cytokine TNF-α, IL-8, and macrophage migration inhibitory factor (MIF), monocyte chemoattractant protein-1 (MCP-1) levels in cells homogenate were analyzed by the corresponding ELISA quantitative kits according to the manufacturer's protocols.

### Dextran sulfate sodium induced colitis in animal experiments

**Figure 1:**
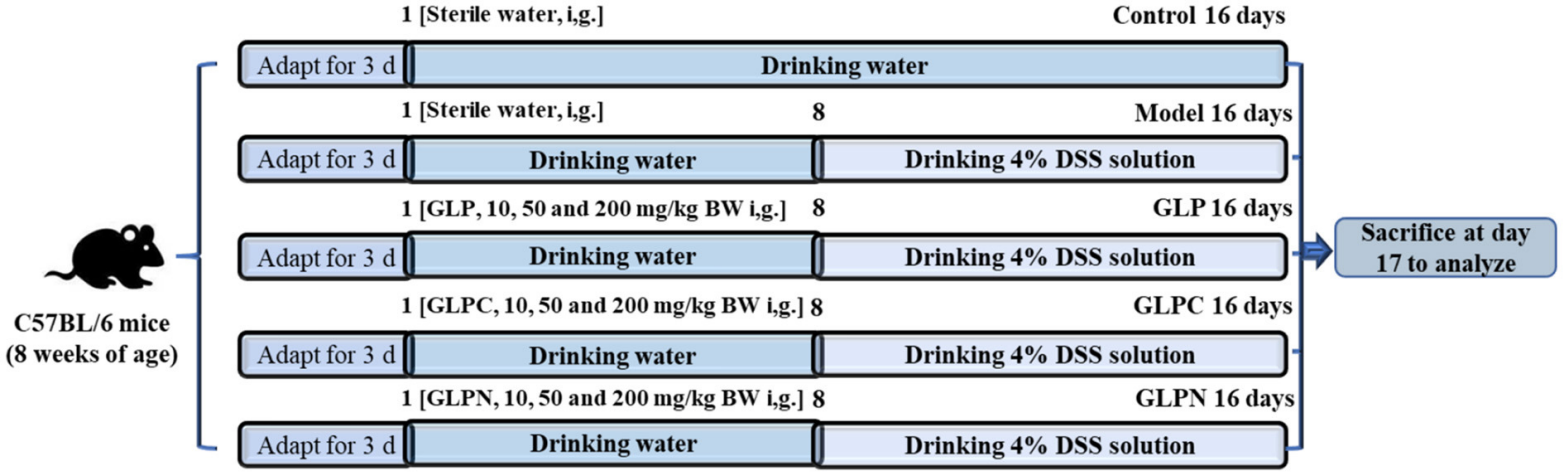
Animal experimental design.

C57BL/6 mice were housed on a 12 h light-dark cycle with free access to water and normal diets. After 1 week of adaptive feeding, they were randomly divided into 11 groups (*n* = 10). The animal experiments were performed as described in the published article (12) with some modifications. The detailed design was shown in Figure 1. The whole experimental period was divided into two periods including pre-treatment with polysaccharides for 7 days (days 1–7) and treatment for another 9 days (days 8–16) with administration of 4% (w/v) DSS solution. During the pre-treatment period, all mice were freely given common drinking water and nine polysaccharide treated groups of mice were administrated by gavage with three different dosages (10 mg, 50 mg, and 200 mg per kg of body weight) of GLP, GLPC, and GLPN, respectively. During treatment period, 4% (w/v) DSS was added into common drinking water for model group and all polysaccharide treated groups of mice, while the manner of polysaccharides gavage remained the same. All groups were named as follows: control group, model group, GLP-L group (10 mg/kg GLP), GLP-M group (50 mg/kg GLP), GLP-H group (200 mg/kg GLP), GLPC-L group (10 mg/kg GLPC), GLPC-M group (50 mg/kg GLPC), GLPC-H group (200 mg/kg GLPC); GLPN-L group (10 mg/kg GLPN); GLPN-M group (50 mg/kg GLPN); GLPN-H group (200 mg/kg GLPN). Body weights of all groups of mice were recorded daily. Finally, the mice were euthanized by cervical dislocation and tissues were collected.

### Assessment of disease activity index

The disease activity index (DAI) was evaluated by scoring the changes of body weight loss, diarrheal condition and fecal bleeding, which is the average score of the three parameters. The scores of each parameter were assigned based on the reported method (26), respectively. Loss of body weight was scored as 0 (no weight loss) and 1–4 (weight loss of 1–5, 5–10, 10–20, and more than 20% from baseline). Diarrhea score were divided into different grades including 0: normal stool; 1: mildly soft stool; 2: soft stool; 3: very soft stool; 4: watery stool; 5: completely watery stool (27). The fecal bleeding score was also divided into 0–5 grades to refer to normal colored stool, brown stool, reddish stool, mildly bloody stool, bloody stool and very bloody stool, respectively (28).

### Evaluation of colonic histopathological score

The colons collected from different groups of mice were gently washed with ice-cold PBS, and fixed in 4% paraformaldehyde for overnight, then embedded in paraffin. After slicing, the colon tissue sections were stained with hematoxylin and eosin (H&E) and examined under the light microscope. The histopathological score was assessed according to the previously reported methods (29).

### RNA isolation and real-time PCR

Total RNA was extracted and isolated from colon tissues using TRIzol lysis (TAKARA Biotechnology Company, Liaoning, China) and used to synthesize cDNA with PrimeScript^TM^ RT Master Mix (Perfect Real Time) Reagent Kit (TAKARA Biotechnology Company, Liaoning, China) according to the manufacturer's instructions. Then mRNA expression levels of GAPDH, TNF-α, IL-1β, and IL-6 were analyzed with quantitative real-time polymerase chain reaction (qPCR) assays using SYBR^®^ Premix Ex TaqTM II (TAKARA Biotechnology Company, Liaoning, China) in the ViiA^TM^ 7 Real-time fluorescence PCR system (Applied Biosystems, USA). The primers of forward and reverse sequences from 5′-3′ used in this experiment were listed in Supplementary Table 1. Data were calibrated to the endogenous reference (*GAPDH* gene) and calculated according to the 2^−ΔΔCt^ method.

### Measurement of pro-inflammatory cytokine levels in colonic tissues

Frozen colonic tissue was homogenized in T-PER^TM^ tissue protein extraction reagent with protease inhibitor cocktail from Thermo at pH 7.6 and extracted for 15 min on ice to obtain tissue protein solutions. Then the extracted solutions were centrifuged, and the supernatant was diluted to the same final concentrations of protein based on the BCA assay (Thermo, USA) analysis. The concentration levels of IL-1β, IL-6, and TNF-α were measured by the corresponding ELISA quantitative kits according to the manufacturer's protocols.

### Statistical analysis

The data were drawn by GraphPad Prism version 5.01 (San Diego, CA, USA) and the results were presented as the mean ± SD. Duncan's multiple-range test and one-way analysis of variance (ANOVA) used to analyze the difference between data from different groups and statistical differences were considered significant and extremely significant at the *P* value < 0.05 and < 0.01, respectively.

## Results and discussion

### Molecular characteristics of β-D-glucan and the degraded fractions

**Figure 2:**
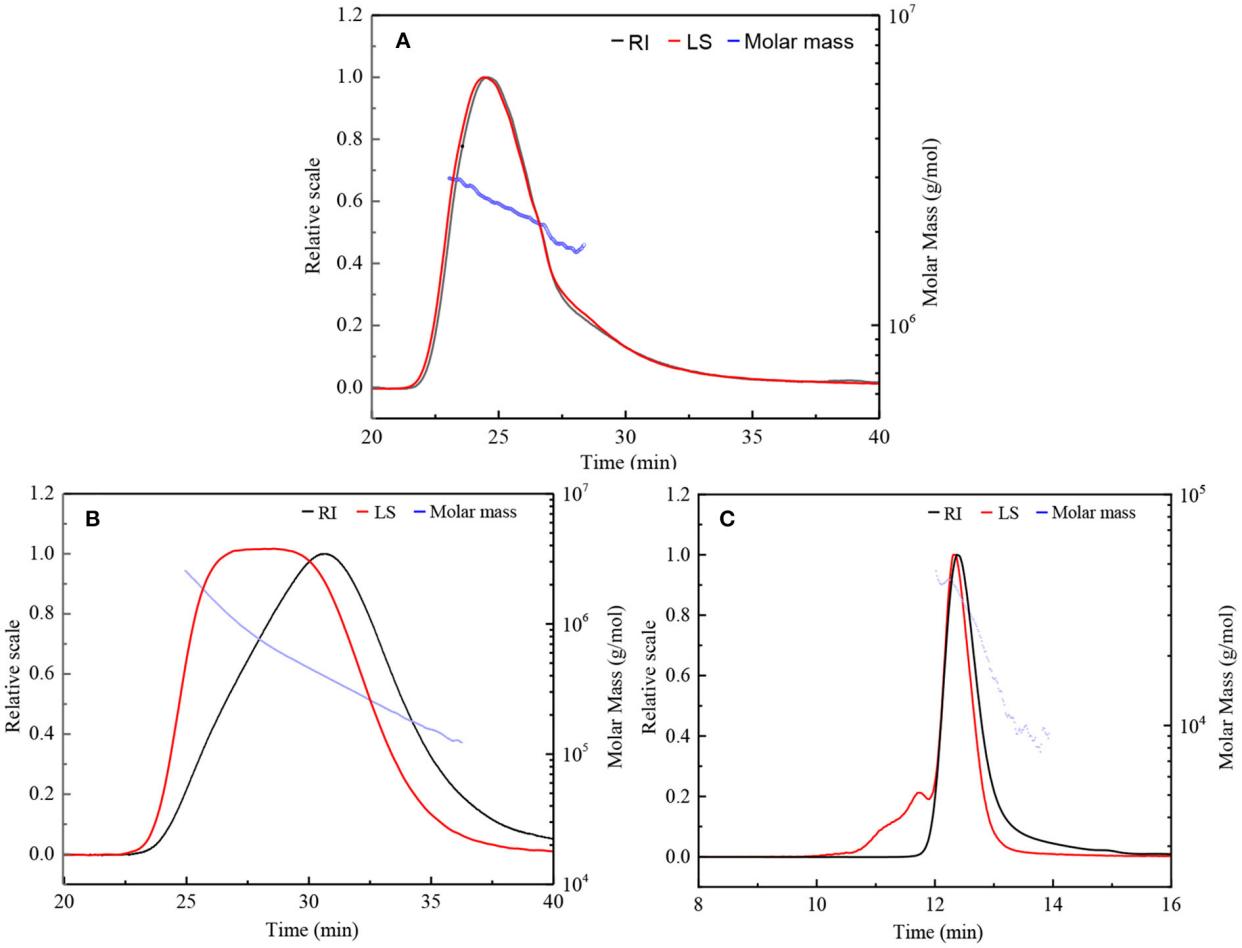
HPSEC elution profiles and molar mass distributions of three fractions. **(A,B)** Represents HPSEC elution profile and molar mass of GLP and GLPC based on columns G6000 PW_XL_ and G4000 PW_XL_ in series connection system, respectively. **(C)** Represent HPSEC elution profile and molar mass of GLPN based on column G2500 PW_XL_ system. RI, differential refractive index; LS, light scattering.

HPSEC eluted differential refractive index (RI) and light scattering (LS) profiles, as well as the molar mass distributions for the original β-D-glucan (GLP) and the ultrasonic degraded fractions (GLPC and GLPN) are shown in Figure 2. The single symmetrical peak occurred in the RI profiles for all fractions, illustrating that ultrasonic degradation could yield homogeneous polysaccharide with the decrease of molecular weight. One single peak detected by LS detector in GLP and GLPC was in agreement with their RI profiles (Figures 2A,B), respectively, indicating no obvious aggregation occurred in GLP and GLPC. Although one single RI peak was detected in GLPN, two peaks detected by LS detector were found in GLPN, indicating some aggregates might be in GLPN solution because the LS signal was more sensitive to large size aggregates than to the concentration of samples (17).

The molecular parameters for all samples including molecular weight (*M*_*w*_), polydispersity index (*M*_*w*_/*M*_*n*_), radius of gyration (*R*_*g*_), hydrodynamic radius (*R*_*h*_), and the ρ-values (*R*_*g*_/*R*_*h*_) were listed in Table 1. The relative low polydispersity index for three fractions (1.32–1.68) revealed that polysaccharide molecules were well-dispersed in the solution. The original β-D-glucan GLP (*M*_*w*_, 2.42 × 10^6^ g/mol) from *G. lucidum* and the sonicated fraction GLPC (*M*_*w*_, 6.53 × 10^5^ g/mol) both exhibited rigid chain conformation with ρ (*R*_*g*_/*R*_*h*_) values higher than 2, which have been proved to exhibit triple helix conformation in aqueous solution (30). For further ultrasonic degraded fraction GLPN, the weight-average molecular weight decreased sharply. The molecular weight distribution analysis of GLPN based on column G2500 PW_XL_ showed that GLPN exhibited a symmetric peak with relatively narrow distribution (Figure 2C) and its *M*_*w*_ was determined to be 3.49 × 10^4^ g/mol. The *R*_*g*_ value of GLPN could not be obtained based on HPSEC-MALLS-RI-VS analysis system due to its low *M*_*w*_, and the *R*_*h*_ value was calculated to be 5.7 nm, which was much smaller than those of GLP and GLPC. According to some researches, the polysaccharide fractions with *M*_*w*_ lower than 1 × 10^5^ g/mol could not form triple helix conformation in aqueous solution (31, 32). Therefore, GLPN exhibited different conformational characteristics with GLP and GLPC.

**Table 1:** Experimental results from HPSEC-MALLS-RI-VS system for β-D-glucan (GLP) and the ultrasonic degraded fractions (GLPC and GLPN) in PBS buffer.

**Samples**	***M_*w*_* (g/mol)**	**Polydispersity (*M_*w*_*/*M_*n*_*)**	***R_*g*_* (nm)**	***R_*h*_* (nm)**	**ρ (*R_*g*_*/*R_*h*_*)**
GLP^a^	2.42 × 10^6^	1.32	166.2	78.5	2.12
GLPC^a^	6.53 × 10^5^	1.68	69.3	33.1	2.09
GLPN	3.49 × 10^4^	1.37	/	5.7	/

a Data of GLP and GLPC were from reference (30).

### Methylation and NMR analysis for polysaccharide fractions

**Figure 3:**
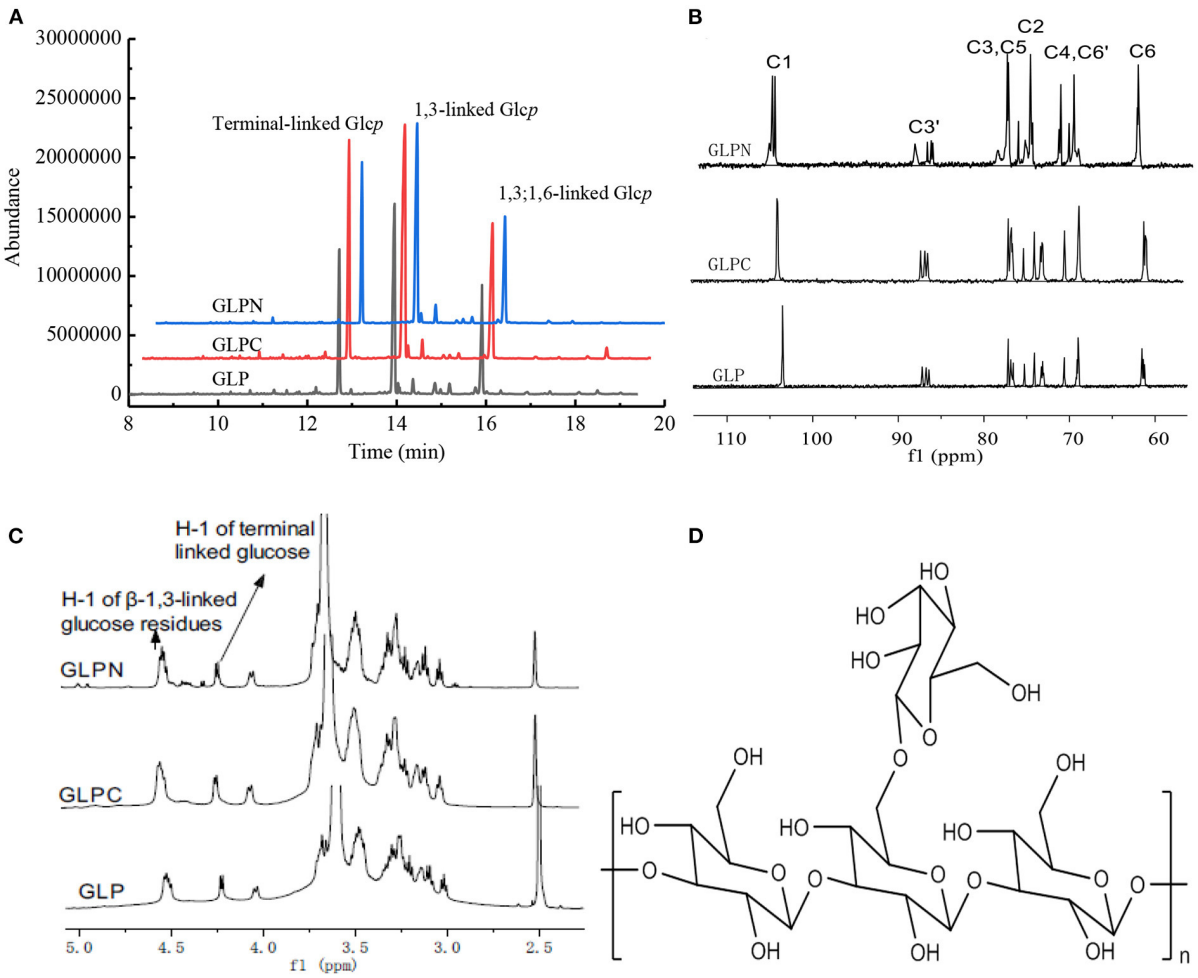
Structure analysis of GLP, GLPC, and GLPN. **(A)** Total ion chromatogram of the methylated products of GLP, GLPC, and GLPN. **(B)** ^13^C NMR spectra of GLP, GLPC, and GLPN. **(C)** ^1^H NMR spectra of GLP, GLPC, and GLPN. **(D)** Primary repeat unit for GLP, GLPC, and GLPN.

In order to elucidate the primary repeat unit of the ultrasonic degraded fractions and confirm if they were similar with that of polysaccharide GLP, the methylation analysis was performed to analyze their linkage types of sugar residues and the corresponding ratios. Total ion chromatograms of the methylated products for GLP and the ultrasonic degraded fractions (Figure 3A) showed that GLPC and GLPN exhibited three obvious peaks, which were the same as GLP and identified as terminal-linked Glc*p*, (1 → 3)-linked Glc*p* and (1 → 3,6)-linked Glc*p*, respectively. The percent molar ratios (Table 2) of three main residues in all fractions seemed similar and were calculated to be about 1:2:1 for terminal-linked Glc*p*, (1 → 3)-linked Glc*p* and (1 → 3,6)-linked Glc*p*, indicating that GLPC and GLPN might possess similar repeat unit with the GLP. The structure features of GLPC and GLPN were further confirmed by ^1^H and ^13^C NMR spectra analysis (Figures 3B,C). Both of the two fractions showed similar peaks as those detected in GLP, suggesting the primary structures for the degraded fractions were not changed. The assignments of protons and carbons were labeled according to the chemical shifts of reported β-D-glucan GLP (21). All three fractions possessed similar repeat unit with different molecular weights and elucidated as β-(1 → 3)-linked D-glucan with a (1 → 6)-D-glucopyranosyl side-branching unit on every third residue (Figure 3D). It has been reported that ultrasonic degradation could produce homologous series of polymers with lower *M*_*w*_ (24) and the structure of polymers could be kept during the degradation process (25). In our study, it was also proved that the two degraded fractions both exhibited similar primary structures with GLP, which could be used to investigate the effects of *M*_*w*_ on the bioactivities of β-D-glucan.

**Table 2:** Methylation analysis of polysaccharide fractions.

**Methylated sugar** **(as alditol acetate)**	**Linkage types**	**Percent molar ratios (%)**			**Major mass fragments** **(*m*/*z*)**
		**GLP**	**GLPC**	**GLPN**	
2,3,4,6-Me_4_-Glc*p*	1-linked-Glc*p*	25.6	27.0	26.2	43, 45, 87, 102, 118, 129, 161, 162, 205
2,4,6-Me_3_-Glc*p*	1 → 3-linked Glc*p*	50.4	48.6	48.9	43, 45, 71, 87, 101, 118, 129, 161, 217, 234, 277
2,4-Me_2_-Glc*p*	1 → 3,6-linked Glc*p*	24.0	24.4	24.9	43, 87, 102, 118, 129, 189, 234, 305

### β-D-glucans inhibited the expression levels of inflammatory cytokines in inflamed Caco-2 cells

**Figure 4:**
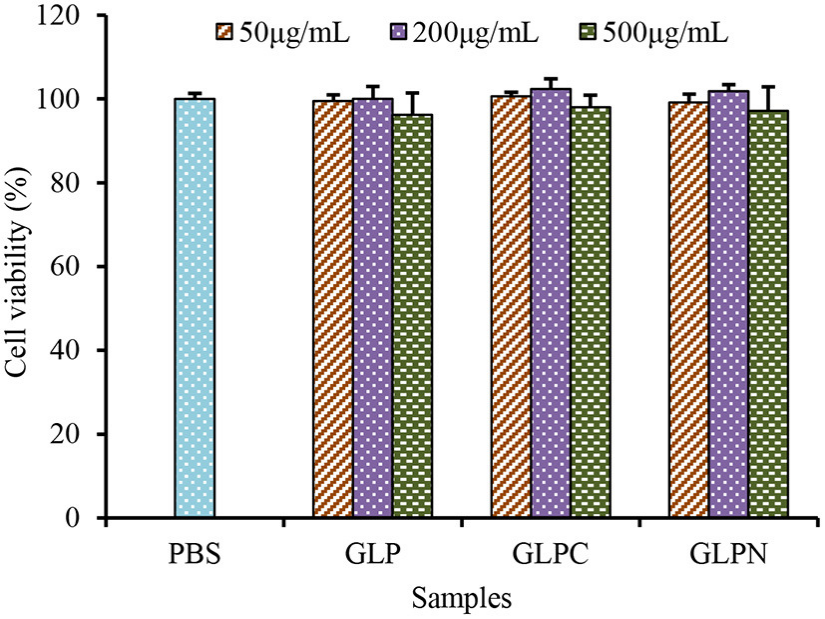
Effects of GLP and the degraded fractions (GLPC and GLPN) on cell viability of Caco-2 cells.

To investigate the effect of β-D-glucans with different *M*_*w*_ on the growth of Caco-2 cells, the cell viability was detected using alamar blue assay after being treated with GLP, GLPC and GLPN for 48 h. Results (Figure 4) showed that the survival rate of cells treated with each fraction (50–500 μg/mL) ranged from 96.21 to 102.34%, indicating that three β-D-glucan fractions had no obvious cytotoxicity on the growth of Caco-2 cells in the tested concentration. So the effects of fractions on the expression levels of inflammatory cytokines in LPS-induced inflamed Caco-2 model could be performed in this range.

**Figure 5:**
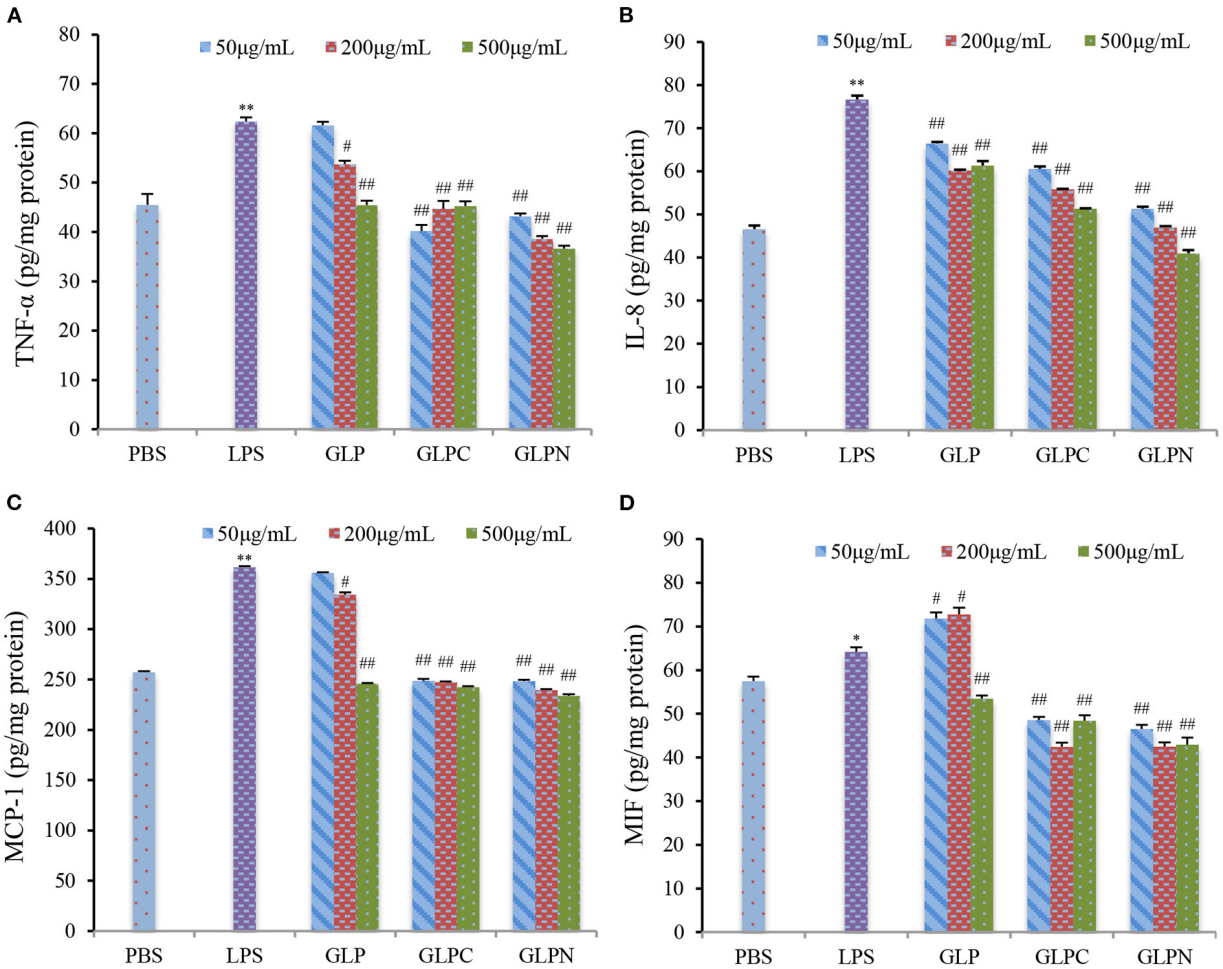
Effects of GLP and the degraded fractions (GLPC and GLPN) on expression levels in Caco-2 cells induced by LPS (2 μg/mL). TNF-α **(A)**, IL-8 **(B)**, MCP-1 **(C)**, and MIF **(D)** expression levels in cells were measured by ELISA. Data are presented as means ± SD (*n* = 3). Significant differences with PBS group were designated as: *P < 0.05; **P < 0.01. Significant differences with LPS-induced group were designated as: ^#^*P* < 0.05; ^##^*P* < 0.01.

It was reported that intestinal inflammatory response was concerted by several cytokines released from epithelial cells (33), which played an important role in the initiation and perpetuation of the inflammatory reaction in IBD. So the selection of inhibitors for cytokines could be an effective therapeutic target in IBD (34). TNF-α is a critical cytokine in the inflammatory process of IBD pathogenesis, and several anti-TNF-α therapies have been proved to effectively reduce pathology in IBD patients (35). Recently, it was found that the macrophage migration inhibitory factor (MIF) also played an important role in the inflammatory process (36). Furthermore, the accumulation of TNF-α and MIF appeared to be pivotal activators of the epithelium to produce pro-inflammatory cytokines such as interleukin (IL)-8 and monocyte chemoattractant protein (MCP)-1 (37). Therefore, TNF-α, MIF, IL-8, and MCP-1 could be thought as inflammatory biomarkers for IBD, and the anti-inflammatory activities can be evaluated by measuring these biomarkers in Caco-2 cells. Our results (Figure 5) showed that the addition of LPS (2 μg/mL) resulted in a remarked increase in TNF-α, MIF, IL-8, and MCP-1 expression levels in Caco-2 cells, while GLP and the corresponding degraded fractions all exhibited significant inhibition on LPS-induced cytokine expression at certain concentrations, indicating that β-D-glucans with different *M*_*w*_ possessed anti-inflammatory potential in LPS-inflamed Caco-2 cells. Among three fractions, the degraded β-D-glucan GLPC (6.53 × 10^5^ g/mol) and GLPN (3.49 × 10^4^ g/mol) exhibited better inhibitory activity compared with the original GLP (2.42 × 10^6^ g/mol), demonstrating that the relatively low molecular β-D-glucans might possess better anti-inflammatory activity in colitis. It was reported that β-glucan from oat with lower molecular weight exhibited stronger activity on reduction of inflammatory markers levels, which might be due to the abundant exposure of glucans in per molar substance to cell receptors in contrast with the fraction with higher molecular weight (38).

### β-D-glucans with different *M_*w*_* ameliorated clinical symptoms in DSS-induced colitis model

**Figure 6:**
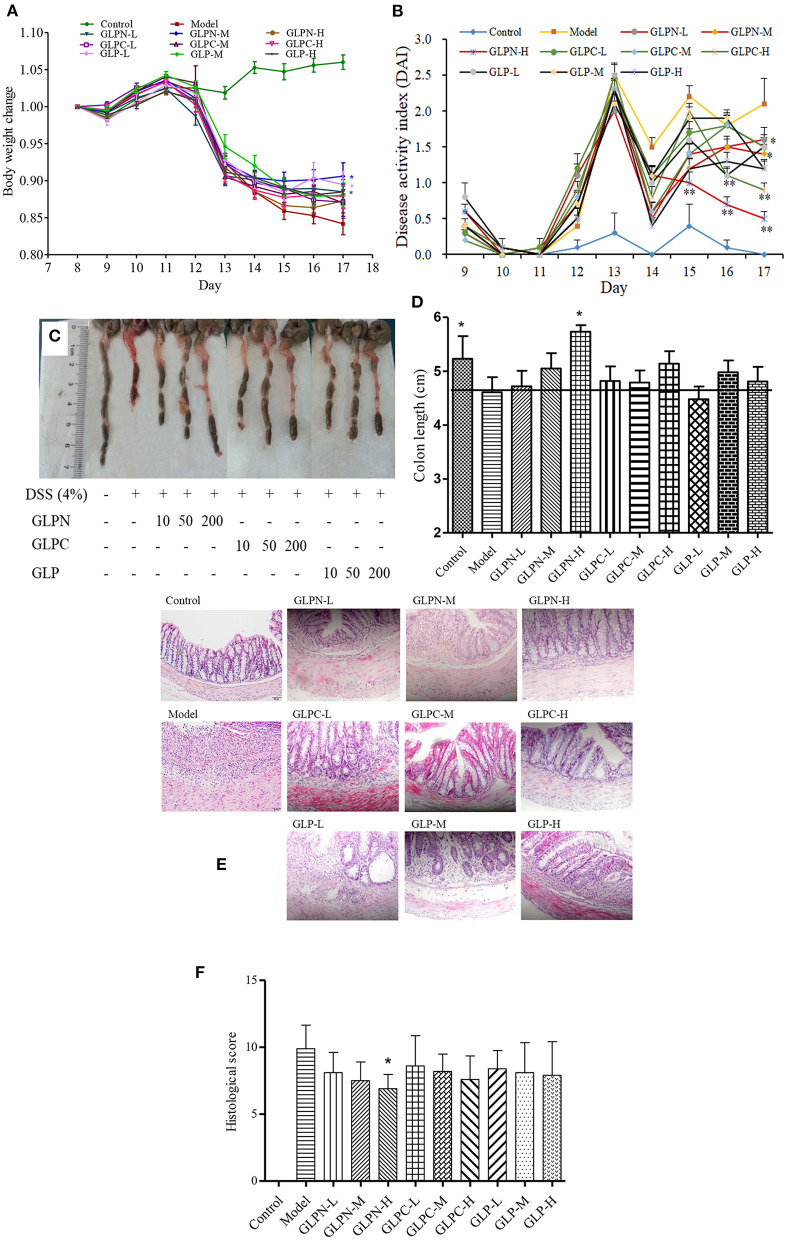
Effect of GLP and the degraded fractions (GLPC and GLPN) on body weight change, disease activity index, and pathological indicators. **(A)** Body weight change; **(B)** Disease activity index; **(C)** The picture of colons in different groups; **(D)** The measured length of colons; **(E)** Representative picture of the colon sections stained with hematoxylin and eosin (H&E); **(F)** Histological score. Data are expressed as mean ± SD, *n* = 10 (*P < 0.05, **P < 0.01 vs. DSS model group).

To confirm the potential therapeutic interventions, DSS-induced colitis animal model is commonly established to study the UC pathogenesis (29). In this study, DSS-induced colitis animal model was applied to compare the anti-inflammatory activity of β-D-glucans with different *M*_*w*_. DSS is a commonly reagent for UC modeling, which can cause symptoms like weight loss and diarrhea. Mice in the model group (DSS-treated group) exhibited significant weight loss than those in the control group from day 13 to 17, whereas oral administration of β-D-glucans could significantly reduce the loss of body weight induced by DSS, especially for GLPN at the dosage of 50 mg/kg (GLPN-M group; Figure 6A). The DAI scores, are usually used to estimate the severity of clinical symptoms in mice with DSS-induced colitis (27). As shown in Figure 6B, the mice treated with β-D-glucans obtained a lower DAI values than those in the model group from day 14 to 17. High dosage of GLPN (200 mg/kg) exhibited extremely significantly lower DAI scores than those of the DSS group, indicating the better anti-inflammatory property on DSS-induced colitis mice.

Additionally, the colon length of mice shortened in DSS-induced model of colitis, which was attenuated by GLPN administration in dose dependence (Figures 6C,D), and high dosage of GLPN (200 mg/kg) exhibited significantly attenuation activity. However, high dosage of GLPC (200 mg/kg) and middle dosage of GLP (50 mg/kg) only presented trends for preventing colon length shortening, indicating that low *M*_*w*_ fraction GLPN (3.49 × 10^4^ g/mol) exhibited better anti-inflammatory effects on DSS-induced colitis in mice. It has been reported that among three exopolysaccharides with high (1.44 × 10^6^ g/mol), medium (9.36 × 10^5^ g/mol) and low (1.97 × 10^5^ g/mol) *M*_*w*_ from *Schizophyllum commune*, oral administration of high *M*_*w*_ fraction could inhibit the shortening of the colon in DSS-induced colitis (39). This was not consistent with the present results and might be due to the different *M*_*w*_ ranges of β-D-glucans for testing.

H&E staining of colon tissues was performed to further analyze the anti-inflammatory of β-D-glucans with different *M*_*w*_. As depicted in Figure 6E, DSS-induced model group displayed severe colonic tissue damage such as inflammatory cell infiltration, lesion formation and crypt destruction compared with control group, while the group treated with β-D-glucans exhibited lightening of colonic damage. The histological score evaluation (Figure 6F) revealed that the lower *M*_*w*_ fraction GLPN significantly reduced the colonic tissue damage and exhibited a pronounced reduction in the inflammatory response and histological scores, indicating that GLPN significantly relieved the symptoms of colonic inflammation. Some researchers reported that oral administration of *Schizophyllum commune* exopolysaccharides with high (1.44 × 10^6^ g/mol) and medium *M*_*w*_ (9.36 × 10^5^ g/mol) possessed intestinal anti-inflammatory activity by recovering inflammation severity. In our results, low *M*_*w*_ fraction GLPN (3.49 × 10^4^ g/mol) was further confirmed to decrease histological score in DSS-induced colitis and exhibited better activity than the other two fractions. The source of β-D-glucans and different *M*_*w*_ ranges of tested samples might lead to different results for investigating the influence of *M*_*w*_ on anti-inflammatory activities of β-D-glucan.

### Effect of β-D-glucans with different *M_*w*_* on suppressing the inflammatory cytokine levels

**Figure 7:**
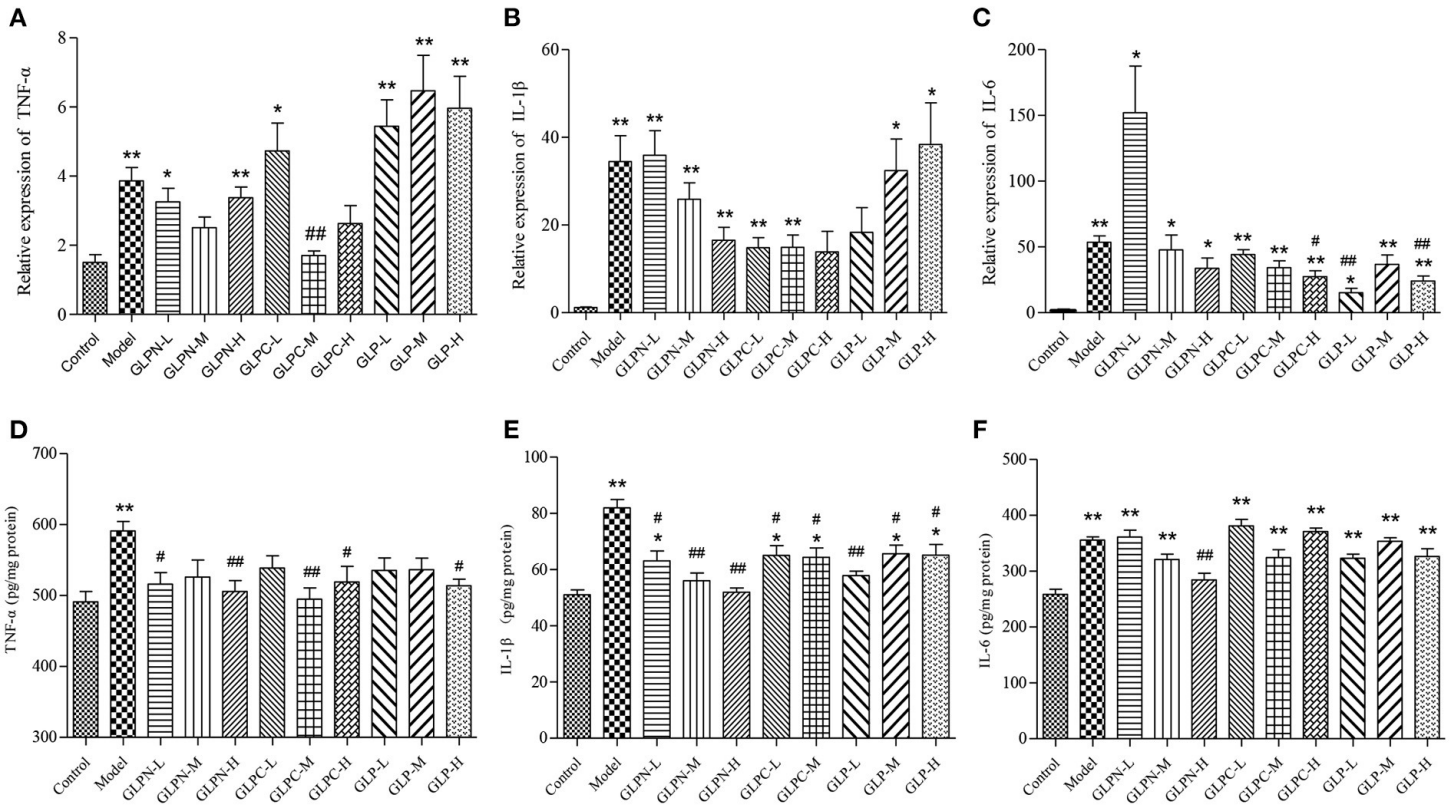
Effects of GLP and the degraded fractions (GLPC and GLPN) on mRNA and protein expression levels of pro-inflammatory cytokines in the colon tissues of different groups of mice. **(A–C)** Represent mRNA expression levels of TNF-α, IL-1β, and IL-6 determined by RT-PCR, respectively. **(D–F)** Represent the expression levels of TNF-α, IL-1β, and IL-6 in colon tissues determined by ELISA, respectively. Data are expressed as mean ± SD, *n* = 10 (*P < 0.05, **P < 0.01 vs. normal control group; ^#^*P* < 0.05, ^##^*P* < 0.01 vs. DSS model group).

Normally the intestinal mucosa with inflammation contains a complex array of inflammatory mediators that can reflect the degree of inflammation. It was reported that these pro-inflammatory mediators played a key role in the pathophysiology of IBD (40). The cytokines (TNF-α, IL-6, and IL-1β) have been implicated as important inflammatory mediators in patients with intestinal inflammation (41, 42). Previous studies have revealed that blockade of these pro-inflammatory cytokines signal in chronic intestinal inflammation caused significant inhibition of colitis (43–45). In the present study, the colon tissues of DSS-induced colitis treated by β-D-glucans with different *M*_*w*_ were performed to test the mRNA and protein expression levels of the inflammatory cytokines (Figure 7). Compared with the normal control group, the mRNA expression levels of inflammatory cytokines in model group were all significantly up-regulated (Figures 7A–C). GLP did not inhibit the up-regulation of mRNA expression levels of TNF-α and IL-1β in the tested dosage, however, it showed obvious inhibition on mRNA expression of IL-6. The degraded fractions GLPC and GLPN could inhibit the mRNA expression levels of TNF-α, IL-6, and IL-1β at a certain dose, indicating the degraded β-D-glucan possessed better anti-inflammatory activity on intestinal inflammation. The expression in protein levels of pro-inflammatory cytokines (Figures 7D–F) also showed that the lower *M*_*w*_ fraction GLPN exhibited extremely significant inhibition on three kinds of pro-inflammatory cytokines at high dosage with 200 mg/kg, which was better than those of GLP and GLPC. The result was consistent with those in inflamed Caco-2 cells model *in vitro*, indicating that GLPN could effectively lessen DSS-induced colitis. These results further confirmed that low *M*_*w*_ β-D-glucan fraction possessed better anti-inflammatory effects.

Some studies showed that the change of *M*_*w*_ could influence the anti-inflammatory activity of polysaccharides. Chang et al. (46) investigated the anti-inflammatory activity of chitosan with different molecular weights *in vitro*, and the result showed that the larger chitosans (*M*_*w*_, 1.56 × 10^5^ g/mol and 7.2 × 10^4^ g/mol) significantly inhibited TNF-α and IL-6 production, whereas the smaller chitosan (*M*_*w*_, 7.1 × 10^3^ g/mol) significantly induced their production (10). Du et al. reported that the triple helical structure of exopolysaccharide from *Schizophyllum commune* would change into random coiled structure by ultrasonic treatment (39). The medium (9.36 × 10^5^ g/mol) and high *M*_*w*_ (1.44 × 10^6^ g/mol) fractions with the conformation of triple helix and single helix showed obvious anti-inflammatory activity in DSS-induced colitis than the low *M*_*w*_ (1.97 × 10^5^ g/mol) fraction with random coiled conformation (39). The study on the effects of oat β-glucan with different *M*_*w*_ in colitis revealed that higher *M*_*w*_ β-glucan showed stronger effects on the reverse in lymphocyte percentage, while lower *M*_*w*_ fraction exhibited stronger suppress effects of the inflammatory markers secretion (38). It was also assumed that high *M*_*w*_ β-glucan might form a protective layer on the internal intestinal wall based on its physical properties, which can reduce inflammatory damages, and low *M*_*w*_ β-glucan was more effective on reducing cytokines through regulating the signal pathways due to its specific molecular structure (38). In our study, the lower *M*_*w*_ fraction (3.49 × 10^4^ g/mol) of β-D-glucan from *G. lucidum* also exhibited significant anti-inflammatory activity than the high (2.42 × 10^6^ g/mol) and middle *M*_*w*_ (6.53 × 10^5^ g/mol) fractions with triple helical conformation structures, especially for reduction on pro-inflammatory cytokines, indicating that decreasing of *M*_*w*_ would improve the anti-inflammatory activity of β-D-glucan from *G. lucidum*. The relationship between *M*_*w*_ and the anti-colitis activity showed difference on polysaccharides with different structures, which might be due to the structural characteristics of these polymers. More fractions with different *M*_*w*_ should be prepared for further investigating the effects of *M*_*w*_ on activities and the deep mechanisms. Our results provided evidence for the potential use of β-D-glucan from *G. lucidum* as a preventive measure for IBD patients.

## Conclusion

In this study, the influence of molecular weight on the intestinal anti-inflammatory of β-D-glucan from *G. lucidum* was investigated. The ultrasonicated fraction GLPC (*M*_*w*_, 6.53 × 10^5^ g/mol) had a similar primary structure and triple helix conformation with the original β-(1 → 3,1 → 6)-D-glucan (GLP, *M*_*w*_ 2.42 × 10^6^ g/mol) from *G. lucidum*, and GLPN (*M*_*w*_, 3.49 × 10^4^ g/mol) also possessed the similar repeat unit as GLP but different conformation characteristics due to its lower *M*_*w*_. The molecular weight showed significant impacts on the anti-inflammatory activity in LPS-induced inflamed Caco-2 cells and DSS-induced colitis model. The degraded fraction (GLPC and GLPN) exhibited better anti-inflammatory activities by inhibiting LPS-induced expression of TNF-α, IL-8, MIF, and MCP-1. Moreover, GLPN with lower *M*_*w*_ fraction exhibited anti-inflammatory activity through preventing colon length shortening and inhibiting the production of pro-inflammatory cytokines including TNF-α, IL-1β, and IL-6 in DSS-induced colitis mice. The results proved that degradation of β-D-glucan could improve its anti-inflammatory activity and contribute to the potential use of β-D-glucan from *G. lucidum* as anti-colitis ingredients.

## Data availability statement

The raw data supporting the conclusions of this article will be made available by the authors, without undue reservation.

## Ethics statement

The animal study was reviewed and approved by the Ethics Committee of Experimental Animal Care at Shanghai University of Traditional Chinese Medicine (Permit No. PZSHUTCM210926015). Written informed consent was obtained from the owners for the participation of their animals in this study.

## Author contributions

YL: conceptualization, funding acquisition, and writing-review and editing. QT: methodology, data curation, investigation, and analysis. JF: methodology, visualization, and investigation. JL: methodology and formal analysis. CT, MY, and JZho: investigation and analysis. SZ: formal analysis, methodology, and investigation. LL: methodology and analysis. JZha: conceptualization, validation, and funding acquisition. All authors contributed to the article and approved the submitted version.

## Funding

This work was supported financially by Shanghai Agriculture Applied Technology Development Program, China (Grant No. X2021-02-08-00-12-F00797), Natural Science Foundation of Shanghai, China (Grant No. 20ZR1418700), Leading Talents Fund in Minhang District of Shanghai, China (Grant No. 201844), and the Project for Excellent Research Team of Shanghai Academy of Agricultural Sciences (Grant No. G2022003).

## Conflict of interest

Author JZho was employed by Shanghai Baixin Bio-Tech Co., Ltd. The remaining authors declare that the research was conducted in the absence of any commercial or financial relationships that could be construed as a potential conflict of interest.

## Publisher's note

All claims expressed in this article are solely those of the authors and do not necessarily represent those of their affiliated organizations, or those of the publisher, the editors and the reviewers. Any product that may be evaluated in this article, or claim that may be made by its manufacturer, is not guaranteed or endorsed by the publisher.

## Abbreviations

DSS: Dextran sulfate sodium
IBD: Inflammatory bowel disease
HPSEC: High-performance size exclusion chromatography
MALLS: Multiple (eight) angle laser light scattering detector
RI: Refractive index detector
VS: Viscosity detector
*M_w_*: Weight average molecular weight
*M_w_*: Number-average molecular weight
*PDI*: Polydispersity index
*R*_*g*_: Radius of gyration
*R*_*h*_: Hydrodynamic radius
TFA: Trifluoroacetic acid
PMAA: Partially methylated alditol acetates
GC-MS: Gas chromatography-mass spectrometry
D_2_O: Deuterium oxide
Me_2_SO-*d6*: Dimethyl sulfoxide-d6
qPCR: Quantitative real-time polymerase chain reaction
ANOVA: Analysis of variance
TNF-α: Tumor necrosis factor-α
IL-1β: Interleukin-1β
IL-6: Interleukin-6
IL-8: Interleukin-8
MIF: Macrophage migration inhibitory factor
MCP: Monocyte chemoattractant protein
DAI: Disease activity index
ELISA: Enzyme-linked immunosorbent assay.
