# Supplementary material for: Effects of molecular weight on intestinal anti-inflammatory activities of β-D-glucan from Ganoderma lucidum
Source: Front Nutr. 2022 Sep 29;9:1028727. doi: 10.3389/fnut.2022.1028727 (PMC9557179; doi:10.3389/fnut.2022.1028727)
Supplement: Supplementary file 1 [file Table_1.docx]

Table S1. Primers for RT-PCR.

| Primers | Primer sequence (5’-3’) |
| --- | --- |
| GAPDH-musF | GGTGAAGGTCGGTGTGAACG |
| GAPDH-musR | CTCGCTCCTGGAAGATGGTG |
| TNF-α-mF | CTGAACTTCGGGGTGATCGG |
| TNF-α-mR | GGCTTGTCACTCGAATTTTGAGA |
| IL-6-mF | TAGTCCTTCCTACCCCAATTTCC |
| IL-6-mR | TTGGTCCTTAGCCACTCCTTC |
| IL-1β-musF | TGCCACCTTTTGACAGTGATG |
| IL-1β-musR | TGATGTGCTGCTGCGAGATT |
